# Survivin can alter mitochondrial architecture by regulating phosphatidylethanolamine synthesis

**DOI:** 10.1242/jcs.263689

**Published:** 2025-08-04

**Authors:** Lucia Dunajová, Amelia Townley, Sophie Rochette, Denise McLean, Jamie R. M. Webster, Sally P. Wheatley

**Affiliations:** School of Life Sciences, University of Nottingham, Queen's Medical Centre, Nottingham NG7 2UH, UK

**Keywords:** Survivin, Mitochondria, Phosphatidylserine decarboxylase, Phosphatidylethanolamine, Phospholipid, Cancer

## Abstract

Survivin (encoded by *BIRC5*) is an essential protein with established roles in mitosis and the inhibition of apoptosis. It is overexpressed in cancers, its abundance correlating with resistance to radiotherapies and chemotherapies. Survivin expression is normally limited to G2 and M phases; however, in cancer cells, it is also present during interphase and gains access to the mitochondria. Phosphatidylethanolamine (PE) is a phospholipid that facilitates negative curvature of membranes. It is enriched in the cytokinetic furrow and mitochondria, where it enables tight packing of the cristae and the increased accommodation of proteins. Here, we report the remarkable discovery that mitochondrial survivin regulates phosphatidylserine decarboxylase activity, thereby affecting PE availability. This novel molecular insight suggests that some apparently disparate roles of this ‘multitasking’ protein might be fundamentally linked to membrane architecture, and offers a new perspective on its contribution to cancer and potentially other metabolic disorders.

## Teaser

**Summary:** Survivin can limit phosphatidylethanolamine availability in mitochondria by inhibiting phosphatidylserine decarboxylase activity.

## INTRODUCTION

Survivin (encoded by *BIRC5*) is a cancer-associated protein that is essential for mitosis and that can inhibit apoptosis (Velculescu et al., 1999; Wheatley and Altieri, 2019). Its expression causes resistance to regular oncotherapies, but exactly how it achieves cytoprotection is not fully understood. In normal somatic cells survivin expression is confined to G2 and M phases; however, in cancer cells, it is transcriptionally upregulated and can be found throughout the cell cycle, and in multiple subcellular compartments, including the nucleus, cytosol and mitochondria (reviewed in Altieri, 2008). To date the mitochondrial pool has only been detected in transformed cells and has been associated variously with greater anti-apoptotic potency (Dohi et al., 2007; Dohi and Altieri, 2005), reduced oxidative phosphorylation (OxPhos) through inhibition of complex I of the electron transport chain (ETC) (Hagenbuchner et al., 2013) and a build-up of defective mitochondria by preventing their clearance via mitophagy (Townley and Wheatley, 2020). However, the full significance of survivin residence in the mitochondrion is incompletely understood, and whether it offers an ‘Achilles heel’ to the cancer field remains an open question (Ausserlechner and Hagenbuchner, 2016).

In order to respire efficiently, the inner mitochondrial membrane (IMM), which houses the ETC machinery, needs to be packed tightly into the mitochondrial volume. Accordingly, cells with high energy demands have many mitochondria with numerous IMM invaginations, known as ‘cristae’, whereas in aged or diseased cells, such as occur in cancer, cardiovascular and neurodegenerative disorders, mitochondria are fewer in number and have reduced cristae density (Fuentes and Morcillo, 2024). Folding of the cristae into the mitochondrion and embedding of the ETC components is maximised by the incorporation of two specialised lipids, cardiolipin (CL) and phosphatidylethanolanime (PE) within the IMM (Fuentes and Morcillo, 2024; Garab et al., 2022). CL and PE have a conical rather than a cylindrical packing modality; thus rather than aggregating within linear lipid bilayers like other phospholipids (Hui et al., 1981), their inclusion forces membranes to curve, which is vital for mitochondrial architecture, function and dynamics (Fuentes and Morcillo, 2024; Garab et al., 2022; Hui et al., 1981).

CL is unique to mitochondria and comprises ∼20% of IMM phospholipids. Owing to its single head with four, rather than two, fatty acid tails, it has a small head-to-tail ratio and conical volume. Its accumulation at the apex of cristae forces the IMM to fold back on itself, which enables the vast IMM to be accommodated (Fuentes and Morcillo, 2024; Garab et al., 2022). PE has a small head but is otherwise a conventional phospholipid with two hydrocarbon tails, and a glycerol-based intermediate. In the mitochondria, PE is made by replacing the serine head of PS with ethanolamine, and as far as has been documented to date, once converted, this pool of PE is used exclusively to sculpt the mitochondrial membranes and is not exported for use elsewhere. Although cytosolic PE is made in the endoplasmic reticulum (ER) via the CDP-ethanolamine or ‘Kennedy’ pathway, mitochondrial PE is made from phosphatidylserine (PS) imported from the ER (Voelker, 1984;^,^ Shiao et al., 1998) through decarboxylation of the serine to ethanolamine, which is accomplished by phosphatidylserine decarboxylase (PSD; also known as PISD) (Vance, 2008; Shiao et al., 1995). Consistent with this, PSD depletion reduces PE availability, compromising mitochondrial IMM integrity, which ultimately impacts metabolic efficiency (Steenbergen et al., 2005;^,^Chan and McQuibban, 2012; Tasseva et al., 2013), cell proliferation and sensitivity to irradiation (IR) (Vance, 2008). Together, PE and CL confer the necessary negative curvature needed for the IMM to pack neatly into the mitochondrion and enable them to maximize ATP production by OxPhos.

Here, we explore the interaction between PSD and survivin in wild-type, non-phosphorylatable (T34A) and phosphomimetic (T34E) forms, and show that its expression impacts PE availability, mitochondrial ultrastructure and respiration in cancer cells.

## RESULTS AND DISCUSSION

**Fig. 1.**
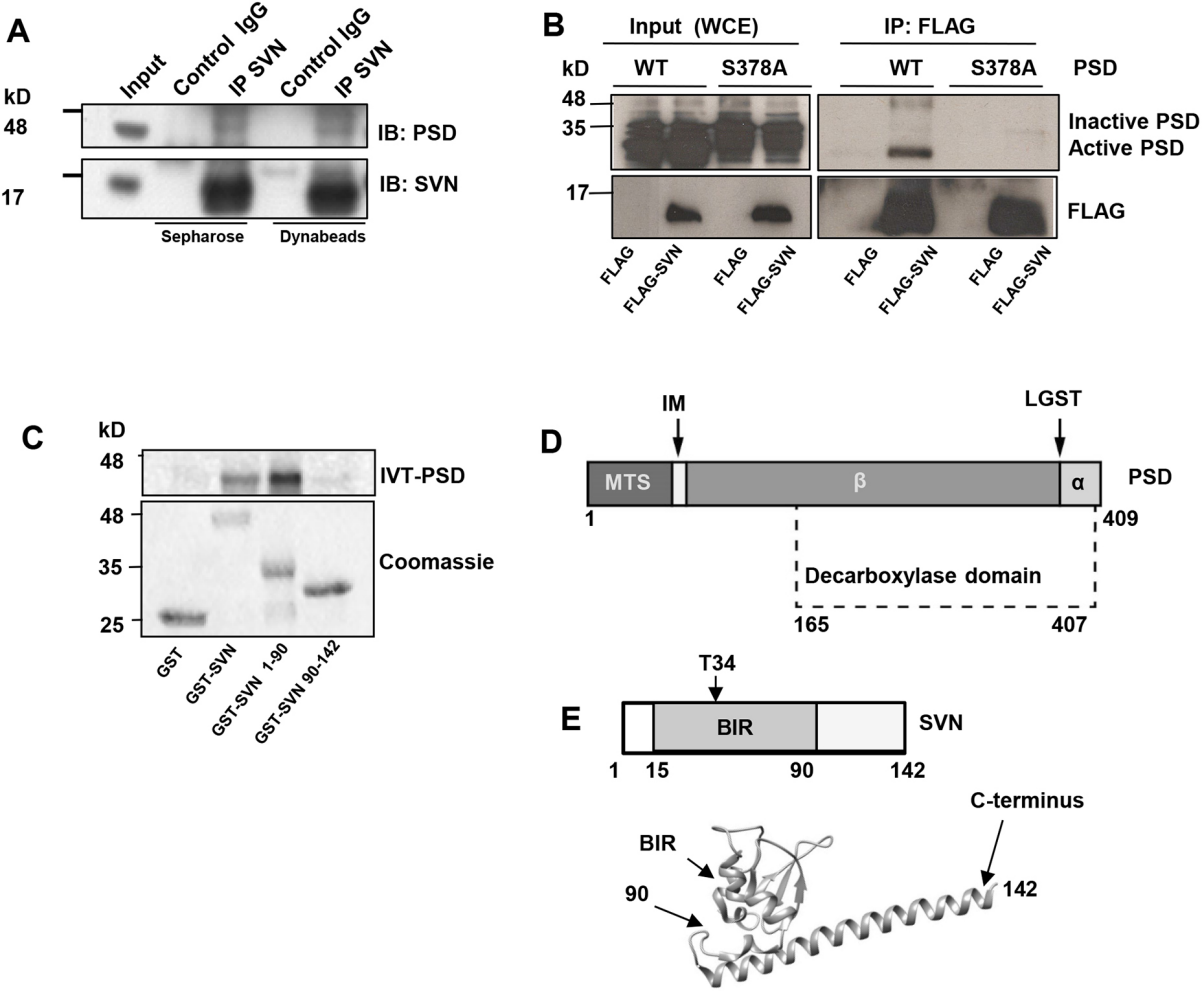
**Survivin interacts directly with active PSD.** (A) Immunoprecipitation of endogenous survivin (SVN) using IgG control and anti-survivin antibodies and sepharose (protein A/G) or dynabeads, followed by immunoblotting with anti-PSD and anti-survivin antibodies. *N*=2, reproduced from Dunajova, 2016. (B) Immunoprecipitation of FLAG and FLAG–SVN from HEK293T cells transiently transfected with the relevant FLAG construct and untagged wild-type PSD (WT) or catalytically inactive PSD (S387A). The immunoblot was probed with anti-PSD and anti-FLAG antibodies. Inputs are 10% total (20 µg) of the whole-cell extract (WCE) and are indicated in left lanes. Representative of *N*=2. (C) *In vitro* pulldown of ^35^S-labelled IVT PSD, expressed from pcDNA3.1 PSD, with GST control, GST-tagged full length survivin (SVN), the N-terminus and BIR domain of survivin (SVN_1-90_) and the C-terminus (SVN_90-142_). Equal loading of GST bait proteins is shown by Coomassie stain. *N*=2, reproduced from Dunajova, 2016. (D,E) Stick models of PSD and SVN highlighting the most salient features. MTS, mitochondrial targeting sequence; IM, inner membrane signal; LGST, autocatalytic motif. Residue numbers are indicated, T34 of SVN is phosphorylated by Cdk1. BIR domain and C-terminus are indicated on crystal structure (taken from PDB 1F3H, and constructed using UCSF-Chimera).

PSD was first suggested to be a survivin-interacting protein after a mass spectrometry screen for novel survivin interactors using survivin as bait and asynchronous HeLa cell extracts (data not shown). To determine whether it was a bone fide interactor, endogenous survivin was immunoprecipitated from HeLa cells using anti-survivin antibodies attached to Sepharose or magnetic dynabeads and immunprecipitates probed with anti-PSD antibodies. As shown in Fig. 1A, PSD co-immunoprecipitated with anti-survivin antibodies but not with control IgG demonstrating that the native proteins interact.

To further validate the interaction and to determine whether survivin interaction depends on the activation state of PSD, the human *PSD* gene was cloned by reverse transcription from total RNA isolated from osteosarcoma (U2OS) cells and subcloned into the mammalian expression vector pcDNA3.1. Transient transfection of pcDNA3.1-PSD into HEK293T cells, in conjunction with pcDNA3.1-FLAG or pcDNA3.1-FLAG-survivin, followed by FLAG immunoprecipitation (IP) and immunoblotting with anti-PSD antibodies (see Fig. S1A,B for specificity) confirmed that PSD co-immunoprecipitated with FLAG–survivin, but not FLAG alone (Fig. 1B). PSD is synthesized as an inactive pro-enzyme that undergoes self-cleavage within the conserved C-terminal tetrapeptide motif ‘LGST’ (Fig. 1D), making two subunits that assemble into the active enzyme (Schuiki and Daum, 2009). An inactive form of PSD was made by replacing the serine residue in this motif with alanine (S378A; see Onguka et al., 2015). Interestingly PSD_S378A_ failed to interact with FLAG–survivin, demonstrating that survivin associates specifically with the active enzyme (Fig. 1B; Fig. S5).

Next, to determine whether this interaction was direct, pulldown assays were performed with immobilized GST–survivin, and ^35^S-labelled PSD *in vitro* translated (IVT) from the pcDNA3.1 vector. Positive interactions were detected between the full-length proteins (Fig. 1C). Subsequent truncation analyses showed that survivin binds to the decarboxylase domain of PSD (Fig. S1C), and the N-terminal 90 amino acids of survivin, survivin_1–90_, which contains the baculovirus inhibitor of apoptosis repeat (BIR) domain, binds to PSD, but the C-terminal domain (amino acids 90–142) does not (Fig. 1C–E).

**Fig. 2.**
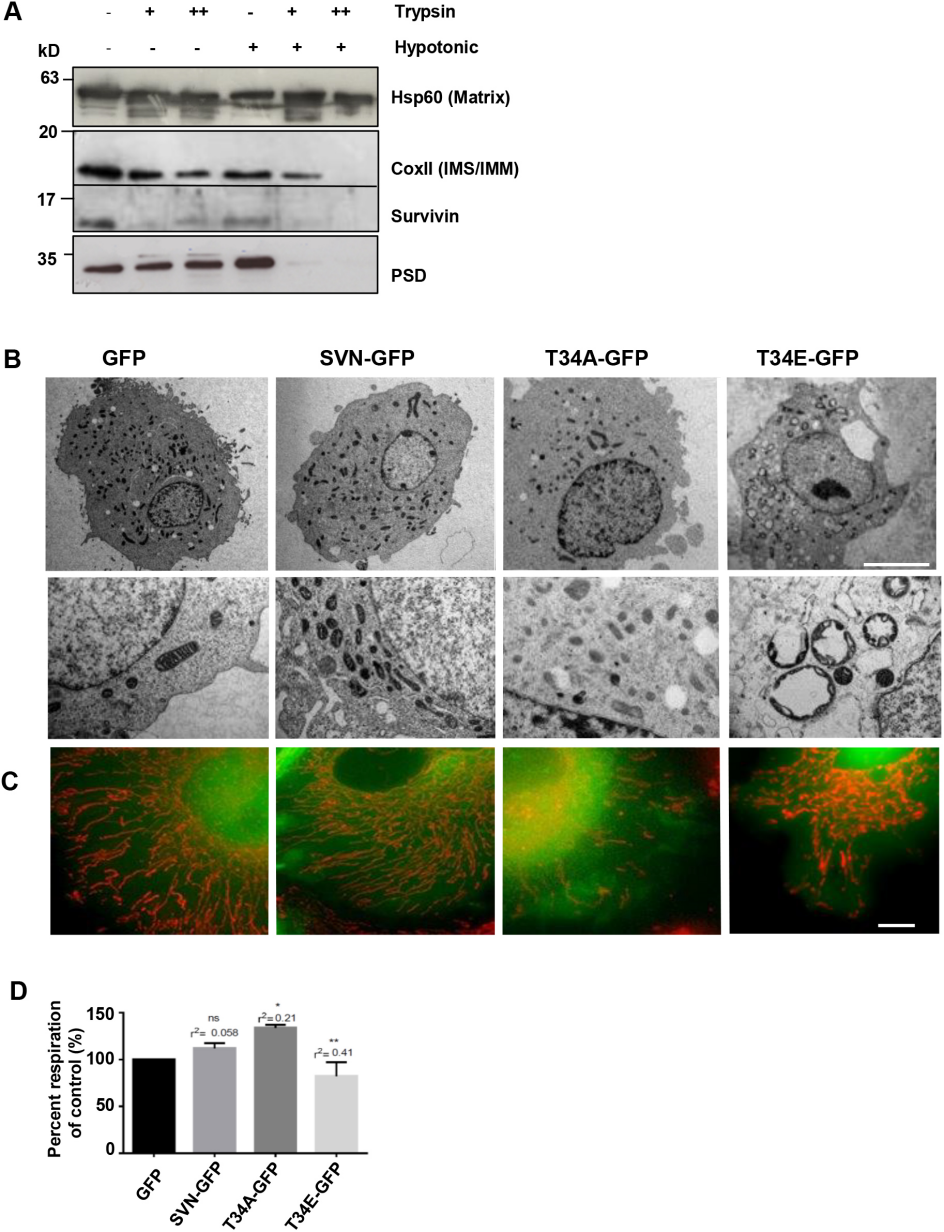
**Expression of survivin mutants causes mitochondrial abnormalities.** (A) Immunoblot of mitochondria isolated from HeLa cells in a protease protection assay treated with trypsin and/or hypotonic shock to investigate whether survivin and PSD are found in the same intramitochondrial compartment(s). *N*=1. Cox II was used to indicate the IMM and IMS, and Hsp60, the mitochondrial matrix. See Fig. S5B for source files. (B) Electron micrographs of mitochondria in HeLa cells expressing GFP or SVN variants. Scale bar: 5 µm. Lower panels show representative magnified images. EM preparations were made two independent times. See Fig. S2A for cell numbers. (C) Representative 2D projected images of deconvolved optical fluorescence *z*-stacks of SVN–GFP (green) variant-expressing cells with mitochondria visualised live using Mitotracker^®^ (red). Images representative of two independent repeats. Scale bar: 10 µm. (D) Respiration was measured for 50,000 cells of each line using the resazurin assay and the mean of *N*=2 independent experiments expressed as a percentage of the GFP control value. *P<0.05; **P<0.005; ns, not significant (unpaired two-tailed *t*-test against the GFP control, r^2^ indicates the magnitude of difference).

Having established that survivin binds directly to active PSD, we next asked whether these two proteins are resident in the same sub-mitochondrial compartment(s). To address this, we used a protease protection assay in which mitochondria isolated from HeLa cells were treated with trypsin before and after osmotic shock, and the remaining proteins were analysed by immunoblotting (Fig. 2A; Fig. S5). Anti-CoxII antibodies were used to identify the IMM and intermembrane space (IMS), and anti-Hsp60 antibodies to detect the mitochondrial matrix (MM). When intact mitochondria were treated with trypsin, in addition to both outer membrane (OMM) markers, PSD and some of the survivin were still present, indicating that at least a portion of each is intramitochondrial, rather than within the OMM. Upon removal of the OMM by hypotonic shock, all proteins in the IMM and IMS were exposed to trypsin, which removed CoxII, PSD and survivin, indicating that they all reside in the IMM and/or IMS, which is consistent with previous reports on the localization of the individual proteins (Shiao et al., 1998, 1995;^,^Dohi et al., 2004). We note that using other methods, survivin has also been seen in the MM (Rivadeneira et al., 2015; Seo et al., 2016), which was not apparent here. However, we are also mindful that our experiment was exploratory (*n*=1), and further investigation is needed to exact its full residency status. Nevertheless, this preliminary result and the literature concur that PSD and some survivin can be found in the IMM.

From the data thus far, we hypothesised that mitochondrial survivin regulates PSD activity in the IMM. In a previous study, two survivin BIR domain mutants that alter the Cdk1-mediated phosphorylation at T34 were generated, survivin_T34A_ and survivin_T34E_, which are non-phosphorylatable and a constitutive phosphomimic, respectively, and expression of which profoundly alter the proliferation rate of human cancer cells and their sensitivity to IR (Barrett et al., 2009). As PSD interacts with the BIR domain of survivin, and the abundance of PE influences growth and the IR response, we proceeded to examine the mitochondrial morphology of cells expressing these mutants. Strikingly the mitochondrial ultrastructure in slow-growing IR-resistant survivin_T34E_ cells was markedly affected – they were highly swollen, lacked internal cristae and had a vast lumen, up to twice the diameter of that in the control cells (Fig. 2B; Fig. S2). By contrast, the mitochondria of survivin_T34A_ cells, which grow rapidly and are hyper-sensitive to IR, appeared ultrastructually normal with dense invaginating cristae. Fluorescence imaging with MitoTracker^®^ revealed that the distribution of the mitochondrial network was also perturbed – appearing varicosed in survivin_T34E_ and diminished in survivin_T34A_ (Fig. 2C), which might reflect hyperfusion and hyperfission, respectively, although the precise dynamics of these networks remains to be rigorously scrutinised (Hagenbuchner et al., 2013, 2016; Adachi et al., 2016). Moreover, when the respiration of these cell lines was compared by seeding equal numbers of cells and assessing reduction of resazurin at 4 h post-adhesion, the rate of respiration was significantly higher in survivin_T34A_-expressing cells (*P*<0.05, *r*^2^=0.21), and lower in survivin_T34E_-expressing cells (*P*<0.01, *r*^2^=0.41), demonstrating that OxPhos is affected (Fig. 2D).

**Fig. 3.**
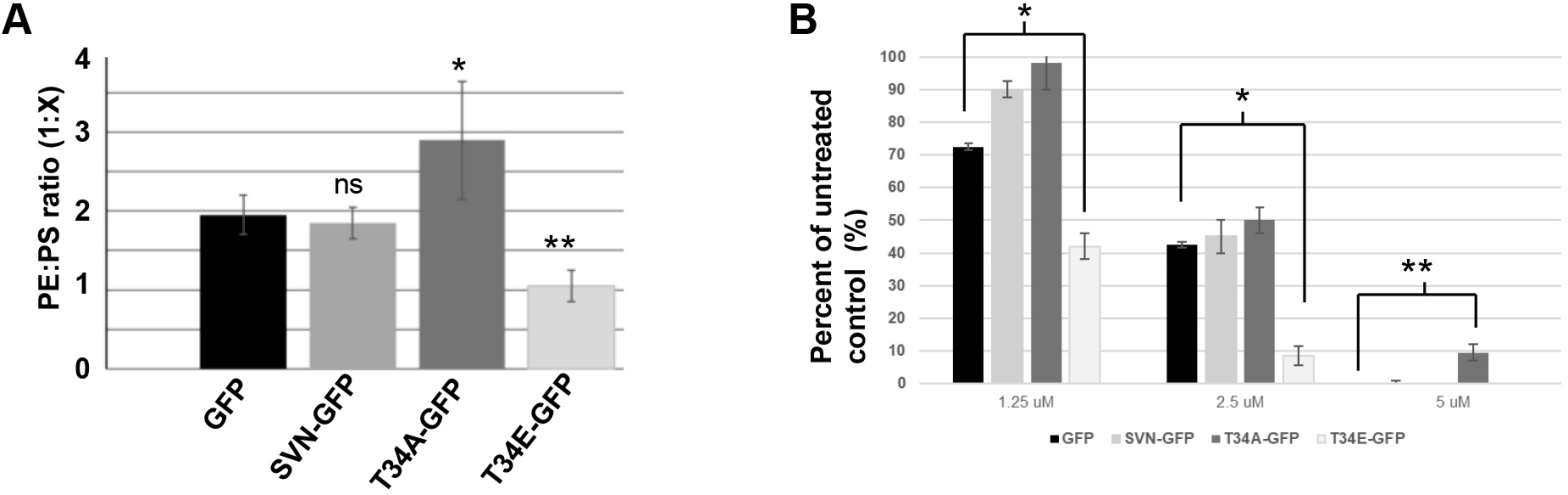
**Survivin regulates PE availability.** (A) Abundance of PS and PE in the stable cell lines indicated was determined by thin layer chromatography (TLC) and plotted as the ratio of PE to PS. Mean of 5 independent repeats, error bars show 95% confidence intervals. A multiple comparison one-way ANOVA was applied. (B) Sensitivity of cell lines to duramycin (48 h). Cell number was determined using a resazurin assay and expressed as a percentage of the untreated control population and a multiple *t*-test was applied (*N*=3); *P*<0.05 is considered significant (*), *P*<0.005 (**); ns, not significant.

To further support the notion that these ultrastructural and metabolic differences could be attributed to altered PE availability, lipids were extracted from these cell lines using phenol-chloroform and analysed by thin layer chromatography. PS and PE were identified by comparison to PS and PE standards and quantified using a phosphorus-based malachite green read-out assay; the abundance of PS and PE was determined for each cell line (Table 1) and the ratio of PE:PS calculated (Fig. 3A). Remarkably, whereas the PE:PS ratio in GFP control cells and those expressing wild-type survivin–GFP was 1.9 to 1, and 1.8 to 1 (PE:PS), respectively, this decreased to 1:1 in survivin_T34E_ cells, demonstrating an accumulation of unconverted PS in these cells. Interestingly the total level of PE did not go down in this extract, which was derived from whole cells; however, this likely reflects contribution of PE generated by the Kennedy pathway to this pool. In stark contrast, the PE:PS ratio in fast-growing IR-sensitised survivin_T34A_-expressing cells increased to 2.9:1, suggesting more efficient conversion of PS into PE. Using IP and cell fractionation, we noted that both survivin_T34A_ and survivin_T34E_ were able to co-immunoprecipitate with PSD (Fig. S3A) and could access the mitochondria (Fig. S3B). From these data, we suggest that the phosphostatus of T34 is an important factor in the regulation of PSD activity by survivin, rather than simply acting to alter binding or mitochondrial entry (see Dunajova et al., 2016).

**Table 1.** Lipid quantification) from whole-cell extracts of lines indicated

	PE	PS	Ratio PE:PS
GFP	12.1	6.2	1.92
Survivin	7.6	4.2	1.8
T34A	14.4	5.0	2.88
T34E	13.3	12.3	1.08

Values are in nmol lipid/mg protein (mean of *N*=4).

To test the PE status of these cell lines further, we treated them with duramycin, a tetracyclic lantibiotic that binds specifically to exposed PE, causing cell lysis in a dose-dependent manner (Iwamoto et al., 2007). Consistent with the lipid analysis, cells expressing survivin_T34A_, which had high levels of PE, were the most sensitive to duramycin, and those expressing survivin_T34E_ were the most resistant to it (Fig. 3B). Although these data lend weight to our hypothesis, PE produced by other biosynthetic pathways (Calzada et al., 2016; Vance, 2008) can be incorporated into the plasma membrane(s); thus we are mindful that duramycin sensitivity might be influenced by these additional pools of PE. Similar to survivin_T34A_-expressing cells, the mitochondria of mouse hepatocytes defective in the enzyme that converts PE into PC, phosphatidylethanolamine *N*-methyltransferase (PEMT), accumulate excessive PE, and are highly metabolically active, generating twofold more ATP via oxidative phosphorylation than PEMT^+/+^ counterparts (van der Veen et al., 2014). We and others have previously shown that overexpression of survivin can cause a metabolic shift to aerobic glycolysis (Hagenbuchner et al., 2013; Townley and Wheatley, 2020), which fits with our hypothesis that survivin_T34E_ limits PE availability and survivin_T34A_ increases it, thus altering IMM ultrastructure and the reliance on OxPhos (Hagenbuchner et al., 2013, 2016). Collectively these data highlight a new and unexpected role for survivin and one that is not part of its repertoire of activities in normal somatic cells. Given the highly collaborative and opportunistic nature of survivin, which as its name suggests, permits cells to adapt and survive inclement conditions, we think it is highly likely that it has additional as-yet-unidentified roles when resident in the mitochondrion.

To begin to address whether it might play additional roles, we depleted survivin or PSD from HeLa cells using siRNA (Figs S4A–C and S5), and compared the mitochondrial to the T34 phenotypes shown in Fig. 2. As shown in Fig. S4A, survivin- and PSD-depleted cells had fewer invaginating cristae and larger luminal spaces than the mock-treated controls (Fig. S4A), which is consistent with previous studies indicating PSD loss causes mitochondrial deformity (Steenbergen et al., 2005;^,^Chan and McQuibban, 2012;^,^Tasseva et al., 2013). However, the phenotype observed was far less acute than witnessed for T34E-expressing cells, suggesting that, at least in the phosphorylated form, survivin could affect additional processes, such as CL integration, within the mitochondria. Nevertheless, when we extracted PE and PS at 72 h post-siRNA treatment, we found that the PE:PS ratio of control cells was ∼2.1:1, whereas survivin depletion reduced this to 1.58:1 and PSD siRNA to 1.83:1 (Table 2), confirming that survivin negatively impacts PE production in these cells.

**Table 2.** Lipid quantification from HeLa whole-cell extracts at 72 h post-transfection with siRNA

	PE	PS	Ratio PE:PS
Untreated	59.3	27.6	2.15
Mock siRNA	68.0	30.4	2.24
Control siRNA	73.1	36.1	2.02
Survivin siRNA	53.7	34.0	1.58
PSD siRNA	54.3	29.6	1.83

Values are in nmol lipid/mg protein (mean of *N*=2).

To conclude, we have shown that by regulating PE synthesis manufactured by PSD, survivin plays a fundamental role in mitochondrial architecture, and that, in a phosphorylated form, this has a significant impact on respiration, which might explain why cells expressing survivin_T34E_ are very slow growing and highly resistant to apoptosis (Barrett et al., 2009). Given the complexities of mitochondrial physiology and function, the importance of PE in membrane structure and the influence of both these parameters on cellular metabolism, this study adds to the ever-increasing list of activities that can be influenced by survivin and has opened a completely new avenue that could be exploited to combat cancer, and potentially other metabolic diseases.

## MATERIALS AND METHODS

### Molecular biology

FLAG-tagged survivin was generated by PCR amplification from pcDNA3.1-survivin (human; see Colnaghi and Wheatley, 2010) using appropriate primers to obtain an insert flanked with BglII sites at both termini. The FLAG tag was placed at the N-terminus. The resulting PCR product was subcloned into pcDNA3.1(+) (Thermo Fisher Scientific). Survivin–GFP constructs have been described elsewhere (Colnaghi and Wheatley, 2010). Full-length wild-type PSD (GenBank accession no. CAG 30426.1) was amplified by PCR from cDNA, generated by RT-PCR from total RNA isolated from U2OS cells. Primers used were: 5′ primer, 5′-GGTAGAATTCATGGCGACGTCCGTGGGGC-3′ and 3′ primer, 5′-GGGCTCGAGCTAGAGCGAGCCCAGGGCTTC-3′. It was subsequently cloned into pcDNA3.1 using flanking EcoRI/XhoI sites. The inactive form, PSD 378A, was created by site-directed mutagenesis using QuikChange (Promega) and the 5′ primer 5′-GTTCAACCTGGGCGCCACCATCGTGCT-3′ and 3′ primer 5′-AGCACGATGGTGGCGCCCAGGTTGAAC-3′. All constructs were verified by sequencing prior to use.

### Cell culture and fractionation

Human embryonic kidney (HEK293), cervical cancer (HeLa) and osteosarcoma (U2OS) cells were cultured at 37°C in DMEM (Sigma D6429) with 10% FCS (Hyclone), and 2 mM glutamine (complete DMEM). All cell lines were originally from ATCC, and were tested for mycoplasma after the experiments were carried out. The medium of cells expressing GFP-tagged constructs was additionally supplemented with 50 µg/ml G418 (Thermo Fisher Scientific). For fractionation analysis, cells were lysed in mitochondrial extraction buffer (10 mM HEPES pH 7.5, 200 mM mannitol, 1 mM EGTA and 70 mM sucrose), in the presence of protease and phosphatase inhibitors (Sigma), by gentle homogenisation with a dounce 2 ml homogeniser. Lysates were centrifuged at 4°C at 1000 ***g*** to remove nuclei, then 10,000 ***g*** to isolate mitochondria. Protein concentration was assessed with a Bradford assay and samples boiled in Laemmli buffer.

### Immunoprecipitations

HEK293T cells (4×10^5^ per well) were transfected with pcDNA constructs using FuGene-6 (Roche) and incubated for 48 h, before harvesting and lysis in co-IP lysis buffer [50 mM Tris-HCl, pH 7.4 (at 4°C), 150 mM NaCl, 1 mM EDTA, 1% Triton X-100]. Lysates were cleared of insoluble debris (12,000 ***g*** for 10 min 4°C) and an ‘input’ sample taken (10%). Anti-FLAG M2 Affinity Gel (Sigma) was added to each sample (30 µl) and incubated with rotation for 2 h at 4°C. The resin was washed thoroughly before being centrifuged and boiled in Laemmli buffer. For the endogenous IP, anti-survivin antibodies (made in house, used as in Colnaghi and Wheatley, 2010) or IgG control antibodies (made in house, used as in Colnaghi and Wheatley, 2010) were used in HeLa cells extracts in conjunction with protein A/G beads (Pierce) or dynabeads (Thermo Fisher Scientific).

### Immunoblotting

Proteins resolved by SDS-PAGE were transferred to 0.22 µm nitrocellulose membrane by standard methods. Membranes were blocked in 5% non-fat milk [w/v in PBS +0.1% Tween 20: PBST; for 2 h at room temperature (RT) or overnight at 4°C] then incubated with primary antibodies diluted in 5% milk for 1 h (RT) or overnight (4°C). Primary antibodies used were against: COXII (1:1000, AbCam, sourced from Lynn Bedford, University of Nottingham, UK), FLAG (M2, 1:1000, Sigma, F1804), Hsp60 (1:5000, gift from Lynn Bedford), PSD (1:500, Sigma, 031090), survivin (1:500, made in house), GFP (1:1000, made in-house), tubulin (1:2000, Sigma, B512) and VDAC (1:1000, Cell Signaling Technology, D73D12). Horseradish peroxidase-conjugated secondary antibodies (DAKO) diluted 1:2000 in 1% non-fat milk for 1 h (RT) were used to detect bands in combination with EZ-ECL chemiluminescence reagents (Geneflow) and Lumifilm (Roche). Blotting source files are shown in Fig. S5.

### GST-tagged protein expression and IVT pulldowns

GST, GST–survivin, GST–PSD and variants of each were expressed in BL21pLysS *E. coli* from pGEX4T1 (Pharmacia) constructs using 0.2 mM IPTG at 37°C. GST-tagged proteins were bound to glutathione Sepharose 4B beads (GE Healthcare) and incubated for 1 h at RT with ^35^S-methionine-labelled (Perkin Elmer) *in vitro* translated survivin or PSD from the relevant pcDNA construct using a T7 TNT^®^ Coupled Reticulocyte Lysate System (Promega). The gels were stained with Instant Blue gel stain (Expedeon) for 20 min, dried and radioactive signals determined using a PhosphoImager and AIDA Image Analyzer software.

### Sub-mitochondrial protease protection assay

Isolated mitochondria (40 µg) were resuspended in control buffer (10 mM HEPES at pH 7.5, 250 mM sucrose) or hypotonic buffer (10 mM HEPES, pH 7.5) for 30 mins on ice, then re-isolated at 10,000 ***g*** for 10 min at 4°C. They were resuspended in control buffer with or without trypsin at 80 or 300 µg/ml (Sigma-Aldrich) for 25 min on ice. Trypsin digestion was stopped with 50 µg/ml AEBSF (Sigma-Aldrich; 15 min on ice). After treatment mitochondria were re-pelleted (10,000 ***g***, 10 min, 4°C) and boiled in sample buffer, or solubilized with Triton X-100 and acetone precipitated prior to boiling.

### siRNA

Survivin was depleted as described in Carvalho et al. (2003). The same method was used for PSD with siRNA from Ambion (ID 118780).

### Lipid extraction

Lipids were isolated from HeLa cells as described by Folch et al. (1957). Briefly, cells were harvested by trypsinization and pelleted at 300 ***g*** for 3 min before resuspending in PBS, aliquoted into three samples and repelleting. One pellet was lysed in HEPES lysis buffer and protein quantified by the Bradford method. The remaining pellets had lipids extracted in 200 μl chloroform/methanol (2:1) per 10^6^ cells. The suspension was incubated for 20 min with shaking at RT before addition of 0.9% NaCl. The samples were then vortexed and centrifuged at 500 ***g*** for 10 min to separate the aqueous and organic phases. The lower organic phase was recovered, vacuum evaporated and resuspended in chloroform.

### Thin-layer chromatography

Chloroform extracted lipids and authentic PS, PC (Avanti) and PE (Sigma) standards were separated on thin layer chromatography (TLC) silica gel 60 plates (Merck) in chloroform, methanol, acetic acid and water 50:30:8:3 (v/v). The plates were dried, and phospholipids visualized with iodine vapour. Silica phospholipid spots were scraped from TLC plates into chromic acid cleaned Pyrex tubes, then digested with 70% perchloric acid at 170°C for 1 h. Phosphate release was quantified using a Malachite Green assay as described in Itaya and Ui (1966). Samples were diluted with ddH_2_O at a ratio of 1:5 perchloric acid: ddH_2_O, and silica gel pelleted (1000 ***g*** for 2 min). Malachite Green solution (0.2%; Sigma) was prepared in ddH_2_O extemporaneously and diluted at a ratio of 15 (Malachite Green solution):5 [4% ammonium heptamolybdate (w/v in 5 M HCl)]:1 (0.5% Tween 20). Samples were incubated for 30 min at RT with 1 ml of 15:5:1 Malachite Green reagent and absorbency measured at 660 nm with a spectrophotometer.

### Fluorescence imaging

To visualise mitochondria, cells were grown overnight on Ibidi 8-chambered microslides, incubated for >15 min at 37°C in CO_2_-independent medium, phenol red-free (Invitrogen) containing 50 nM MitoTracker^®^ Red CMXRos (Invitrogen). Cells were imaged with a 60× (NA1.4) objective using a IX71 Olympus Delta Vision Elite microscope operated with Applied Precision software. 3D stacks (0.3 µm slices) were acquired and presented as 2D TIFF projections, prepared in Adobe Photoshop and ImageJ.

### Electron microscopy

Cells were washed in 0.1 M cacodylate buffer, incubated for 1 h with 1% osmium tetroxide in 0.1 M cacodylate buffer, then washed with distilled water and dehydrated with a graded ethanol series of 50, 70, 90 and 100% ethanol, followed by immersion in 100% propylene oxide, 3:1 and 1:1 propylene oxide:resin, respectively. Cells were infiltrated with epoxy resin overnight then embedded and polymerized at 60°C for 48 h. Ultrathin sections (80 nm) were cut with a diamond knife using a Leica EM UC6 ultra microtome, placed on copper grids, and then analysed using a Tecnai G12 Bio-TWIN transmission electron microscope (FEI Company), run at an accelerated voltage of 100 kV. Images were captured using a MegaView SIS camera.

### Duramycin treatment

The GFP-tagged stable HeLa cell lines (5×10^3^ cells) were seeded in 96-well plates, allowed to adhere for 3 h and medium changed to complete DMEM supplemented with 50 µg/ml G418 supplemented and either 0 μM, 1.25 μM, 2.5 μM or 5 μM duramycin (Acros Organic). Cell number was assessed by incubation with 10 µg/ml resazurin in DMEM for 1 h at 37°C. Absorption was measured spectrophotometrically (FLUOstar Galaxy, BMG Labtechnologies) with excitation and emission spectra at 530 and 590 nm. The experiment was repeated three times.

### Statistical tests

All statistical analysis was performed using Prism 7 (GraphPad) software; details of tests are given in figure legends.

## Supplementary Material

10.1242/joces.263689_sup1 Supplementary information
