# Supplementary material for: Survivin can alter mitochondrial architecture by regulating phosphatidylethanolamine synthesis
Source: J Cell Sci. 2025 Aug 4;138(15):jcs263689. doi: 10.1242/jcs.263689 (PMC12377709; doi:10.1242/jcs.263689)
Supplement: Supplementary information [file joces-138-263689-s1.pdf]

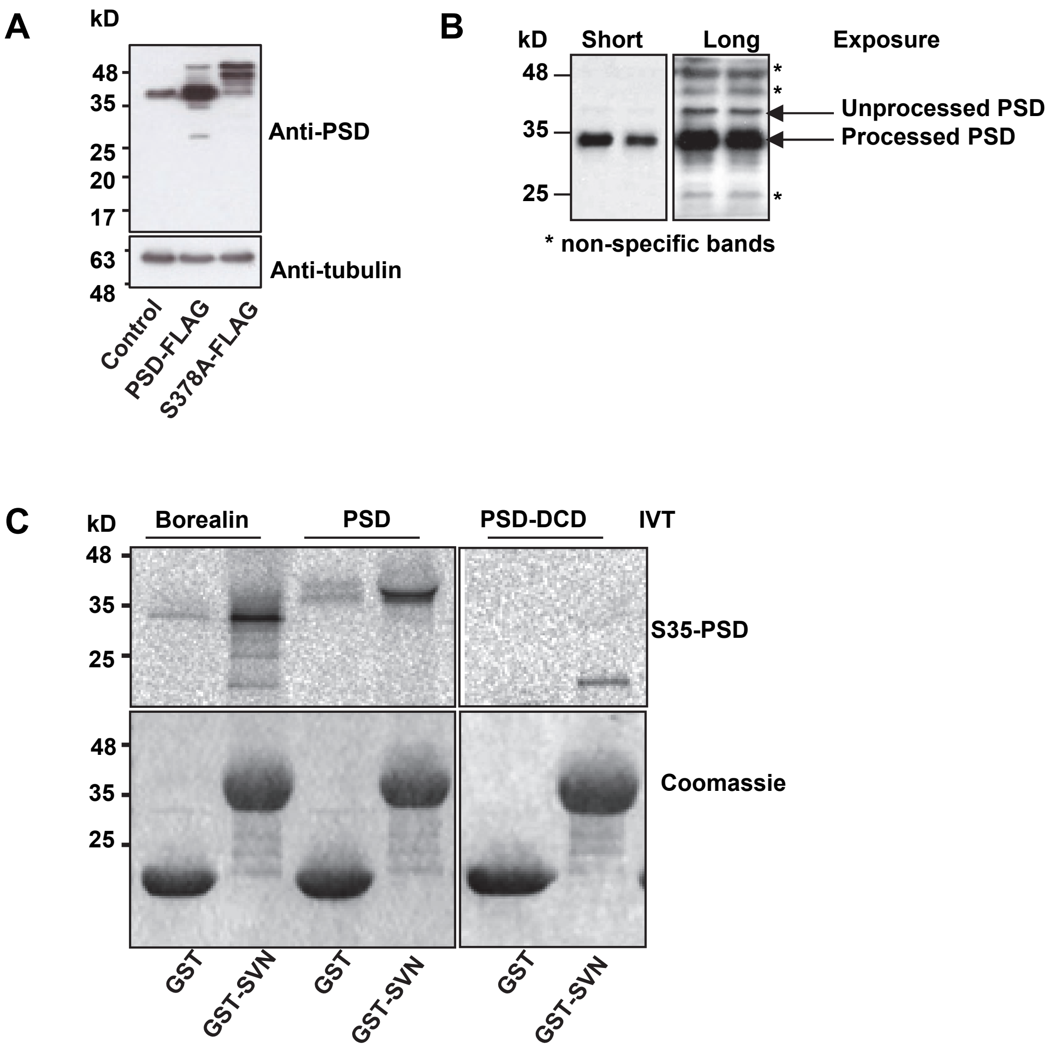

Fig. S1. (A) Immunoblot analysis with anti-PSD antibodies to demonstrate its specificity against endogenous PSD (control lane), ectopically expressed FLAG tagged PSD, and the inactive FLAG tagged form, in whole cell extracts; tubulin given as loading control. N=1. (B) Short and long exposures as in (A) control lane to demonstrate antibody specificity. N=1. (C) *In vitro* pull down of ^{35}S-labelled IVT PSD or its decarboxylase domain (DCD), expressed from pcDNA3.1, with GST control, or GST-SVN. Borealin was used as a positive control. Equality of GST loading is revealed by Coomassie stain. N=2.

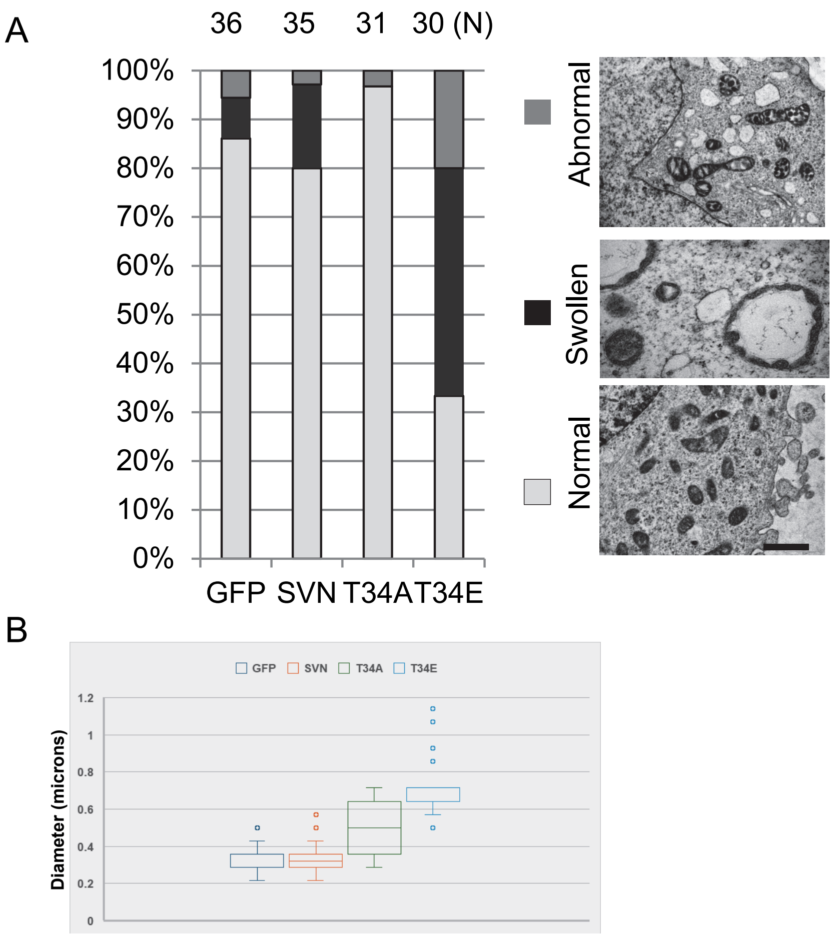

Fig. S2. (A) Quantitation of mitochondrial morphologies seen by EM, expressed as a percentage of the total number of cells (N) viewed, categorised as normal, swollen or abnormal. (B) The mean diameter of mitochondria (+/-SD) is plotted for N>/= 20 mitochondria per sample. EM preparations were made 2 independent times.

Journal of Cell Science • Supplementary information

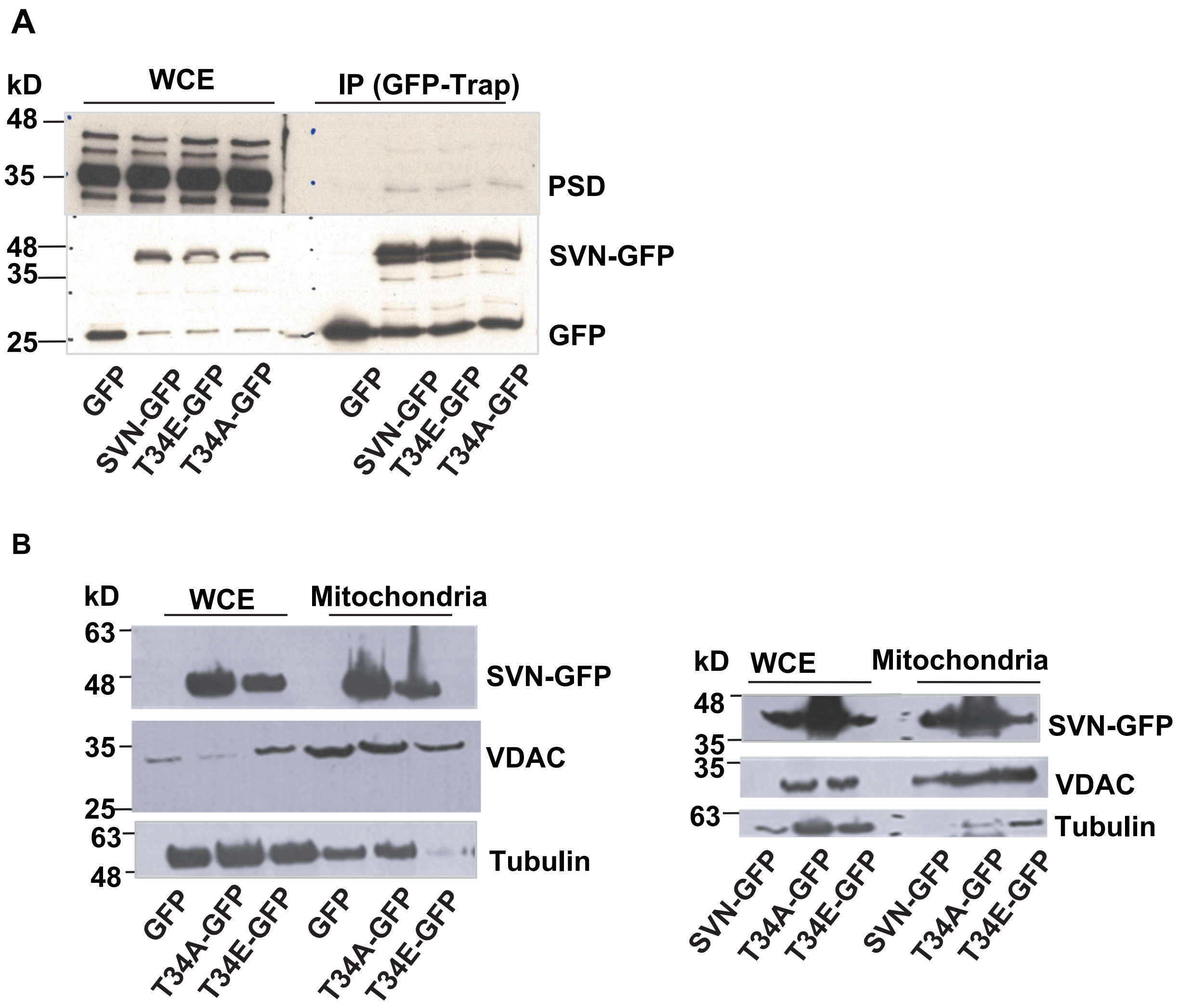

Fig. S3. (A) Co-immunoprecipitation of endogenous PSD was carried out using GFP-trap after transient transfection of HEK cells with cDNA to GFP or the SVN variant indicated (note order of lanes; GFP expression alone not shown), and untagged PSD, as detailed in[23]. N=2. See also Figure S5C. (B) Mitochondrial fractionation carried out as described in[23] on stable HeLa lines expressing GFP or SVN variants. Mitochondrial enrichment was assessed using anti-VDAC antibodies; tubulin indicates any cytosolic contamination in mitochondrial samples. N=2. See also Figure S5D.

Journal of Cell Science • Supplementary information

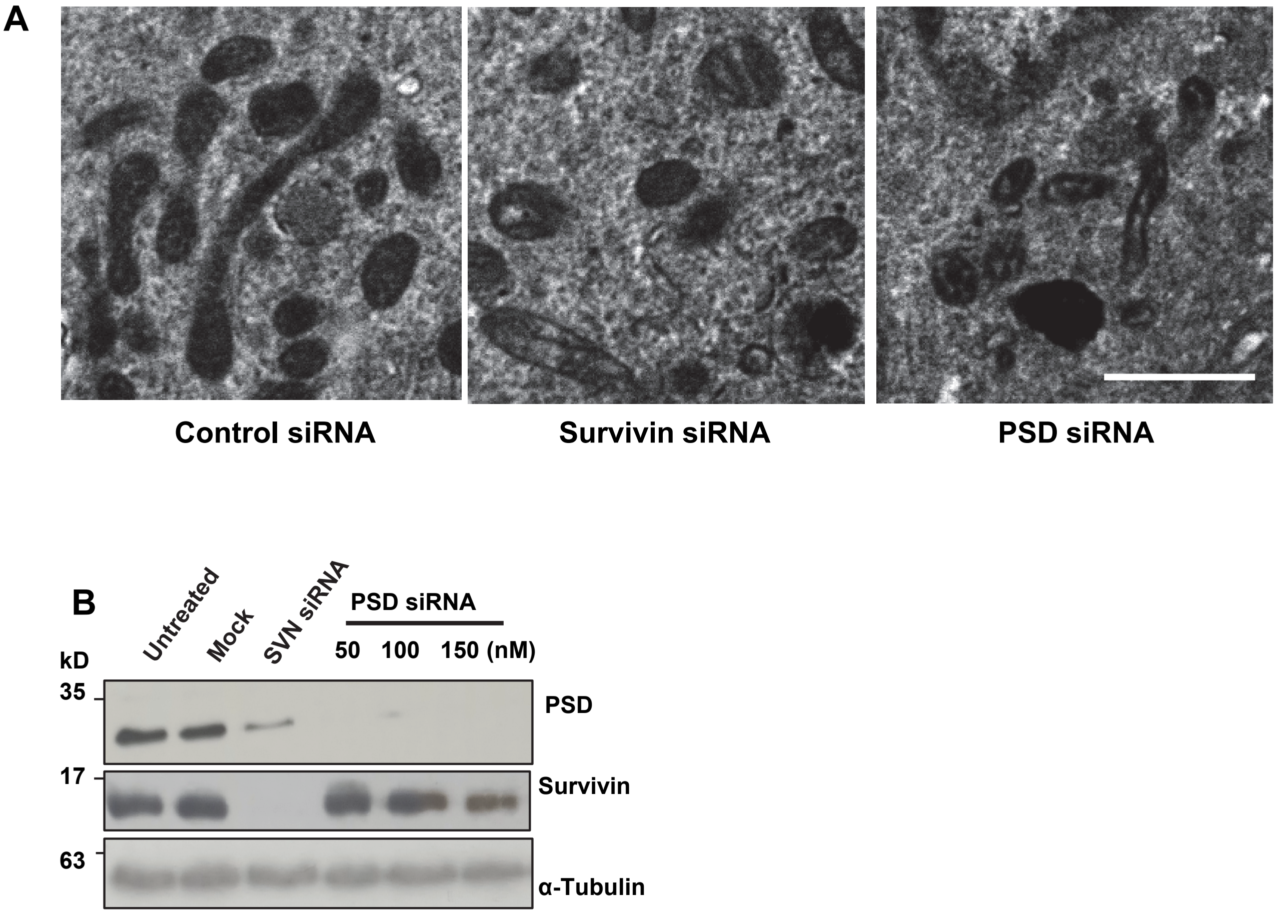

Fig. S4. (A) siRNA depletion: EM images of mitochondria in cells treated with mock (transfection reagent alone), 100 nM survivin or PSD siRNA. Bar 1 μm. (B) Immunoblot to demonstrate efficacy of knock down using 50-150 nM PSD siRNA and survivin siRNA (72h). Tubulin indicates equality in loading. N=3. See also Figure S5E.

A. Source file for Figure 1B

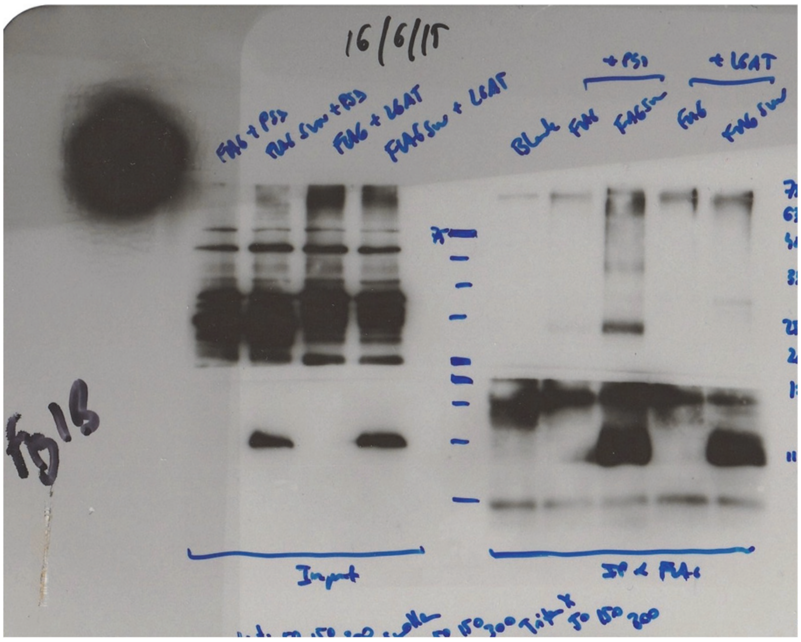

B. Source files for Figure 2A – first 6 lanes are used in the main figure

Long exposure

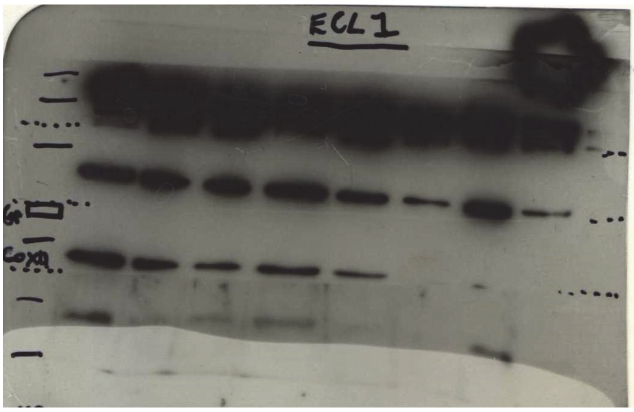

Anti-Hsp60

Anti-VDAC, not used

Anti-CoxII

Anti-survivin

35

25

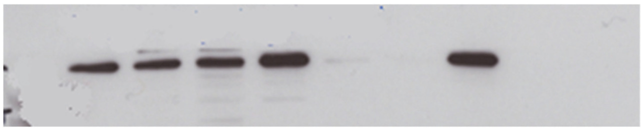

Anti-PSD

Short Exposure

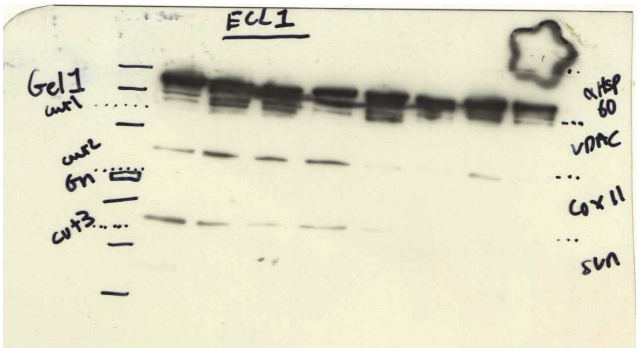

Anti-Hsp60 version used

Journal of Cell Science • Supplementary information

C. Source files Figure S3A

Whole cell extract IP (GFP-trap)

Anti-PSD

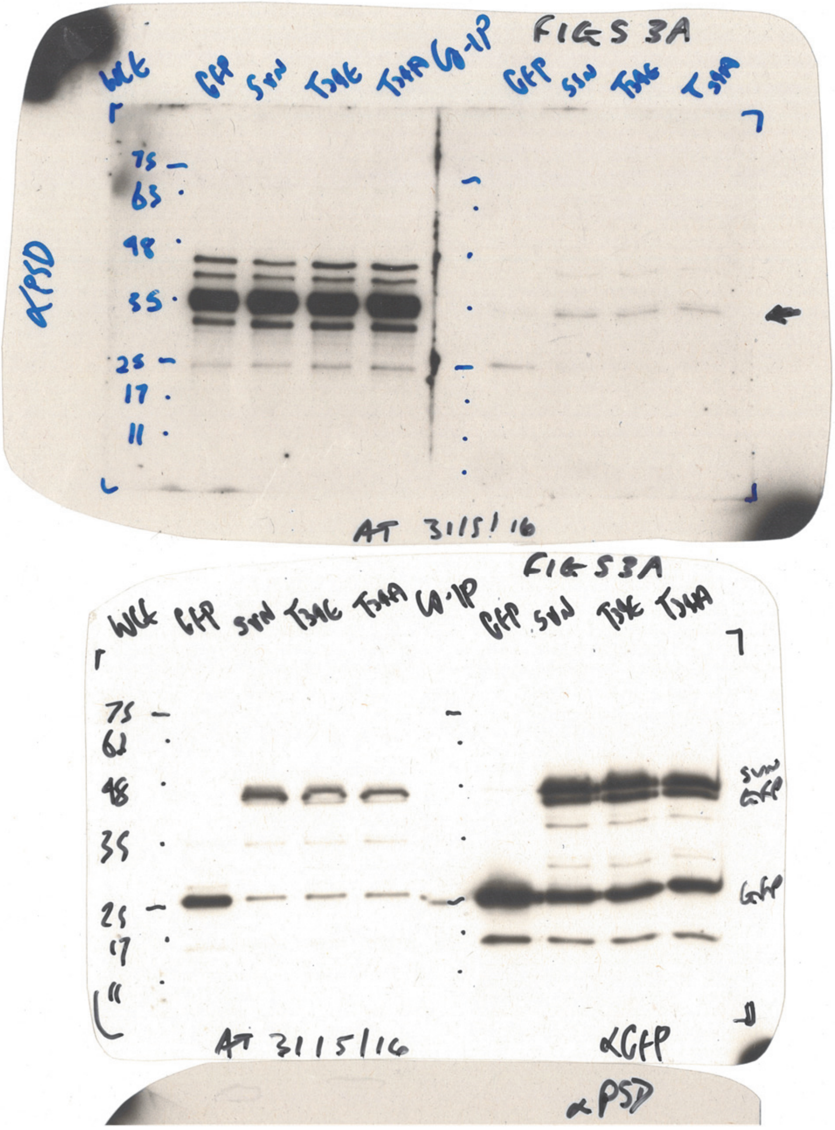

Anti-GFP

GFP SVN T34E T34A GFP SVN T34E T34A

Note order of the lanes

Journal of Cell Science • Supplementary information

D. Source files Figure S3B

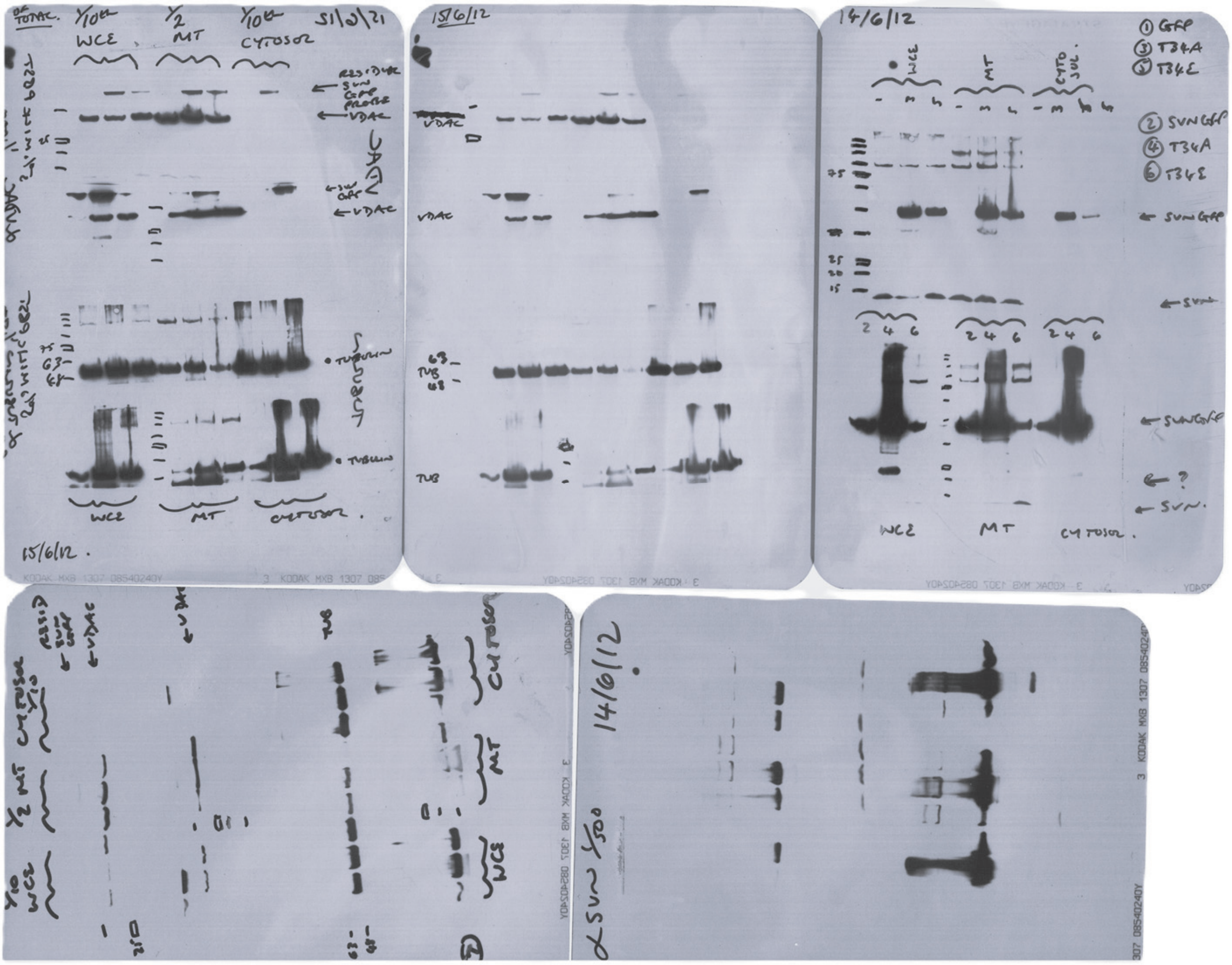

Second Repeat

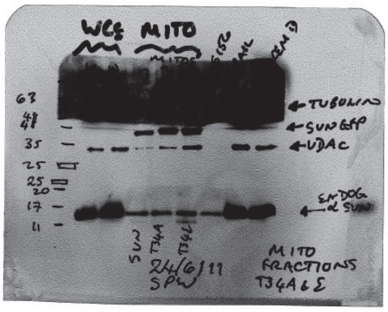

E. Source files for Figure S4 – siRNA –

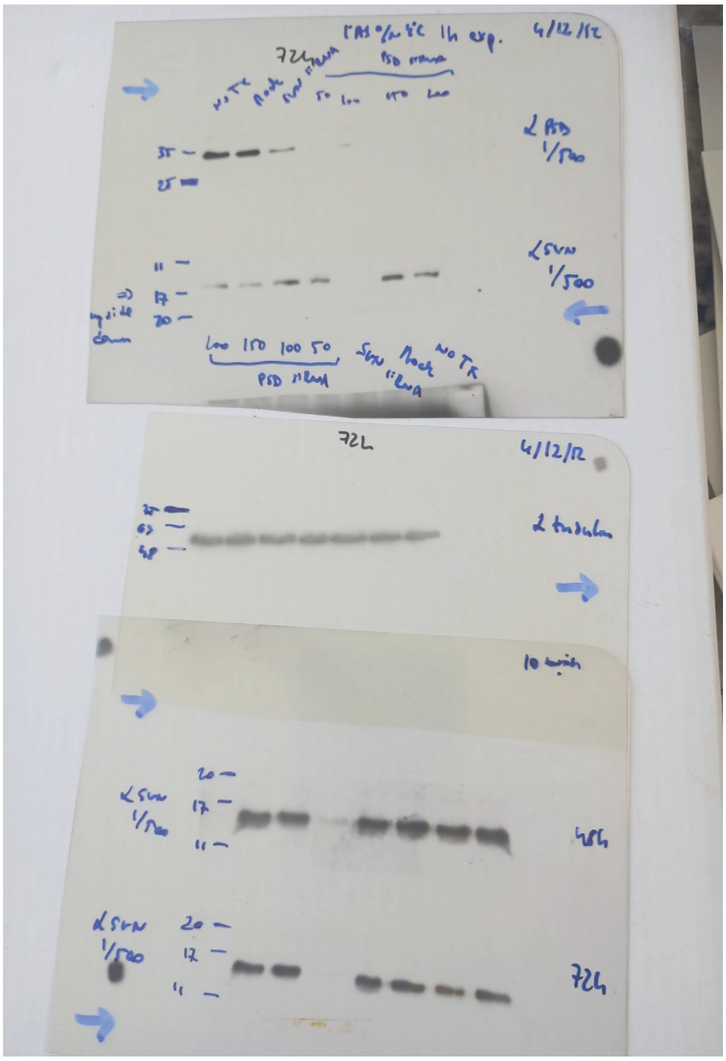

Additional siRNA blot (48h)

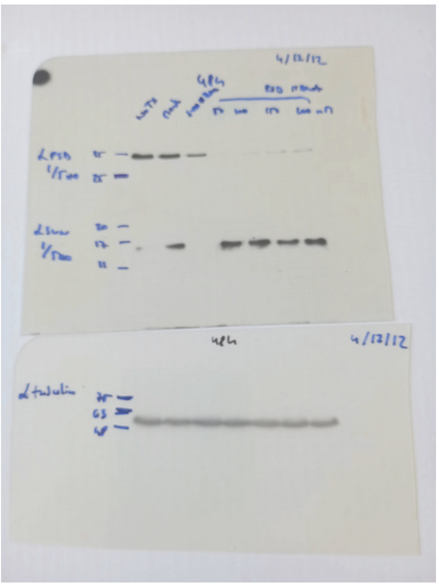

Fig. S5. Blot transparencies
